# An Atypical *Kappa*‐Class Chaperone‐Usher Fimbriae of a Human Enterotoxigenic *Escherichia coli* Strain Shows Multi‐Host Adherence and Distinct Phylogenetic Feature

**DOI:** 10.1111/1348-0421.13208

**Published:** 2025-03-02

**Authors:** Hiharu Inoue, Yoshihiko Tanimoto, Dongming Zheng, Erika Ban‐Furukawa, Miyoko Inoue, Yuko Omori, Yoshihiro Yamaguchi, Taro Tachibana, Hisashi Aso, Weiping Zhang, Eriko Kage‐Nakadai, Yoshikazu Nishikawa, Takayuki Wada

**Affiliations:** ^1^ Graduate School of Human Life and Ecology, Osaka Metropolitan University Osaka Japan; ^2^ Institute for Life and Medical Sciences, Kyoto University Kyoto Japan; ^3^ Graduate School of Human Life Science, Osaka City University Osaka Japan; ^4^ Graduate School of Science, Osaka Metropolitan University Osaka Japan; ^5^ Graduate School of Engineering, Osaka Metropolitan University Osaka Japan; ^6^ Cellar Biology Laboratory, Graduate School of Agricultural Science, Tohoku University Sendai Japan; ^7^ Department of Pathobiology University of Illinois at Urbana‐Champaign Urbana Illinois USA; ^8^ Faculty of Human Sciences, Tezukayama Gakuin University Osaka Japan; ^9^ Osaka International Research Center for Infectious Diseases, Osaka Metropolitan University Osaka Japan

**Keywords:** adhesion, Chaperone‐usher fimbriae, colonization factors, comparative genomics, enterotoxigenic *Escherichia coli*

## Abstract

The pathogenesis of enterotoxigenic *Escherichia coli* (ETEC) involves the colonization of hosts by colonization factors (CFs) and the secretion of enterotoxins. CFs, especially chaperone‐usher fimbriae, mediate bacterial adhesion to host cells, with extensive genetic diversity observed among isolates. One ETEC strain, O169YN10, possessed a unique plasmid (pEntYN10) encoding three CFs, CS6, and two novel homologs of CS8 and F4 (CS6_O169_, CS8_O169_, and F4_O169_). In this study, F4_O169_ was found to play a major role in adhesion to multiple hosts, including human, bovine, and porcine epithelial cells, whereas the other two CSs were less functional. Inhibition assays using antibodies showed that FayG1, one of the two major paralogous adhesins of F4_O169_, directly contributes to human cell adhesion. Despite the established function of FayG1, the FayG2 protein was not detected under the in vitro conditions. Comparative genomics revealed that FayG1 and FayG2 share low homology with other *E. coli* strains isolated from hosts, suggesting sporadic emergence from an unknown origin.

**Abbreviations**
AA: amino Acid
BIE cells: bovine intestinal epithelial cells
CF: colonization Factor
CFU: colony‐forming unit
CS: coli surface antigen
CU: Chaperone‐Usher
DMEM: Dulbecco's modified eagle medium
ETEC: enterotoxigenic *Escherichia coli*
FBS: fetal bovine serum
HEp‐2: human epidermoid larynx carcinoma cell line‐2
IPEC‐1: intestinal porcine epithelial cell‐1
LB: Luria‐Bertani
MEM: minimal essential medium

## 1 Introduction

Colonization of the intestinal epithelia, mediated by colonization factors (CFs) or coli surface (CS) antigens (adhesins), is an essential initial step in the pathogenesis of enterotoxigenic *Escherichia coli* (ETEC) [1, 2]. These bacteria generally adhere to host cells as an initial step and subsequently secrete either or both heat‐labile and heat‐stable enterotoxins, causing diarrheal condition [3, 4]. In contrast to the conserved peptide sequences of enterotoxins of ETEC strains from the main reservoirs (human, porcine, and bovine), CFs exhibit an abundance of variations that orient the target of adhesion, which may be associated with host tropism [1, 5]. Extensive study of CFs precedes the insights gained from genetic/phylogenetic classifications. Notably, human specific ETEC CFs have been numbered in accordance with their discovery, starting with the letter “CFA (colonization factor antigen)” or “CS (coli surface antigen).” Meanwhile, fimbrial structures on the surfaces of bacteria discovered until then had been numbered with the letter “F,” irrespective of genetic relatedness [6].

Chaperone usher (CU) fimbriae, the most diverse genetic family of CFs, plays a pivotal role in the attachment of *E. coli* to its host cells [7, 8, 9]. Furthermore, the structure on the surface of bacilli mediates their adherence to host cells, which is a crucial step in the establishment of infection. CU fimbrial operons exhibit extensive genetic and antigenic diversity, with a vast array of variants reported across different ETEC isolates [10]. Enteroaggregative attachment found in some pathogenic *E. coli* strains is also known to be mediated by CU fimbriae, such as *aaf1‐5* and CS22 [11], which are well‐known adhesins responsible for the adhesion of enteroaggregative *E. coli*. Moreover, investigations into the properties of CU fimbriae as CFs, including the molecular mechanisms of ligand recognition essential for adherence, are considered critical targets for controlling ETEC infections. Importantly, several studies have highlighted the potential for developing a vaccine against ETEC that utilizes CU fimbriae as an antigen [12, 13, 14]. These observations encourage further exploration of the relationship between the genetic diversity of CU fimbriae and host interactions, which could offer valuable insights into bacterial adaptation and host‐pathogen dynamics.

O169YN10, a strain exhibiting serotype O169:H41, was first isolated as an ETEC from foodborne cases in Japan [15]. Notably, the serotype strains have been sporadically identified as causes of foodborne infection not only in Japan but also in the US and South Korea [16, 17, 18, 19, 20]. In a detailed study, it was observed that plasmid pEntYN10 could provide its host strain with adherence to HEp‐2 cells in a unique enteroaggregative manner [21]. Genetically, the ETEC strain was verified to possess a gene encoding STp (heat‐stable toxin type Ia), a variant of STs typically found in pigs, the causative enterotoxin of human diarrhea [22], and CS6, a typical CF of STp‐possessing ETEC [23]. Uniquely, the strain often loses its adhesive activities with great ease after several sequential passages in vitro [15], probably owing to the loss of its virulence plasmid, which appears to help the bacteria sustain its ability to infect the host by retaining it.

To explain the peculiar nature of this strain O169YN10, the complete sequence of its unstable plasmid, pEntYN10, was determined in a previous study [24]. The plasmid consists of approximately 150 kb, encoding the STp gene and three operons of CFs: CS6 with minor variants and two unreported homologs of CS8 (CFA/III) and F4 (K88) [24]. Although CS6 of the strain was very close in sequence to the known subtypes, the uniqueness of the remaining two CFs was outstanding. In particular, the homologous fimbria of F4 (F4_O169_ hereinafter) possesses two paralogous major adhesin genes (*fayG1* and *fayG2* hereinafter) at the 3′ end of the operon. This is inconsistent with the typical *kappa* (*K*) class CU fimbriae [8] to which F4 belongs, in which only one major adhesin subunit gene is encoded at the middle position of the operon. Secondly, in the homologous operon of CS8 (CS8_O169_ hereafter), CofA showed high amino acid sequence identity (73.2%) with the major fimbrial subunit of CS8 [24]. Owing to the voracious coexistence of CFs, it remains unknown which CFs are responsible for adhesion to human cells as virulence factors in the ETEC strain.

In this study, we demonstrated that the F4 homolog of pEntYN10 (F4_O169_) plays a major role in the adhesion of multi‐host epithelial cells in vitro. Bacterial adherence inhibition experiments using antibodies against the two adhesins showed that FayG1 directly contributes to adhesion to human cells. Furthermore, based on comparative genomics using bacterial genome sequences retrieved from a database, it seems that adhesins emerged sporadically without similar sequences in *E. coli*, evoking another question regarding their origin.

## 2 Materials and Methods

### 2.1 Bacterial Strain

ETEC strain O169YN10 was originally isolated from a diarrheal patient [15]. The strain lacking its plasmid (pEntYN10) was obtained by multiple passages of culture, as shown in Ban et al. [24]. The *E. coli* laboratory strains TOP10 were purchased from Takara Bio Inc., Shiga, Japan. All bacterial strains were cultured in Luria‐Bertani (LB) broth (Becton, Dickinson and Co., NJ, USA) at 37°C. When necessary, chloramphenicol was added to the media at a concentration of 170 µg/mL.

### 2.2 Recombinant Strains

The three CFs operons encoded in pEntYN10 (F4_O169_, CS6_O169_, and CS8_O169_) with both ends of the untranslated regions (ca. 1000 bp, including the original promoter and terminator) were amplified by polymerase chain reaction (PCR) using Takara Ex Taq DNA polymerase (Takara Bio), with the purified plasmid as a template. The PCR primers are listed in Table 1. The pSTV28 vector (Takara Bio) was digested using EcoRI‐HF and BamHI‐HF (New England Biolabs, MA, USA). Purified PCR products and linearized vectors were ligated using an In‐Fusion Cloning Reaction Kit (Takara Bio). The resulting plasmids (pSTV28‐F4_O169_, pSTV28‐CS6_O169_, and pSTV28‐CS8_O169_) were recovered by transformation into *E. coli* TOP10 with selection for chloramphenicol resistance. After plasmid purification, Sanger sequencing was performed using primer walking to ensure that there were no mutations in the cloned sequence. The primers are listed in Table S1.

**Table 1:** List of PCR primers used in this study.

Target gene	Primer name	Oligonucleotide sequence (5′–3′)	Product size (bp)
F4_O169_ fragment 1	K88‐1.2‐F^a^	CCATGATTACGAATTCCTTACACCCTTCAACATCGG	6181
	K88‐1.2‐R	ATTGCTCTGAAGATGCCACT	
F4_O169_ fragment 2	K88‐1.6‐F	CATCTTCAGAGCAATCTTGG	6147
	K88‐1.6‐R^a^	CGACTCTAGAGGATCCTCATCATCAGTAAGGGAACG	
CS6_O169_ fragment 1	CS6‐1.5‐F^a^	CCATGATTACGAATTCTGAGTCGTCTGGAGCATTAT	3712
	CS6‐11.5‐aR	GCATGGATCCCGTTATCTAT	
CS6_O169_ fragment 2	CS6‐1.5‐bF	TAACGGGATCCATGCTTTAT	3663
	CS6‐1.5 R^a^	CGACTCTAGAGGATCCATATTCACGGTGATCCACAC	
CS8_O169_ fragment 1	cof‐1.0‐aF^a^	CCATGATTACGAATTCTCTTACTCCATCCAGGTCAA	4875
	cof‐37‐R	CGCTGGTCAGAACCAGATTA	
CS8_O169_ fragment 2	cof‐37‐F	TGGTTCTGACCAGCGGACTT	5464
	cof‐2/3‐R	TGCATCCACTCCATCATATT	
CS8_O169_ fragment 3	cof‐2/3‐F	GATGGAGTGGATGCAAATTA	4706
	cof‐1.0‐bR^a^	CGACTCTAGAGGATCCACGTTTCTGGTGCCATGATC	

^a^ The common sequences of the 5′ end of primers (CGACTCTAGAGGATCC and CCATGATTACGAATTC) were common to the in‐fusion cloning reaction.

### 2.3 Cell Lines

HEp‐2 cells were grown in minimal essential Eagle's medium (MEM; Nissui, Tokyo, Japan) containing 2 mM l‐glutamine, 0.15% NaHCO_3_, 1× nonessential amino acids for MEM (MP Biomedicals, CA, USA), and 10% fetal bovine serum (FBS; Biosera, Nuaille, France). Caco‐2 cells were grown in MEM containing 2 mM l‐glutamine, 0.15% NaHCO_3_, 1 mM sodium pyruvate (MP Biomedicals), 1× nonessential amino acids for MEM, and 10% FBS. Porcine epithelial cells (IPEC‐1) were grown in Dulbecco's Modified Eagle Medium (DMEM)/Ham's F‐12 (Fujifilm Wako Pure Chemical Corp., Tokyo, Japan) containing insulin–transferrin–selenium premix solution (MP Biomedicals), 5 ng/mL Epidermal Growth Factor (Fujifilm Wako Pure Chemical Corp.), and 5% FBS. Bovine intestinal epithelial (BIE) cells were grown in DMEM (Nissui) supplemented with 1 mM sodium pyruvate and 10% FBS. Cells were grown in 25‐cm^2^ polystyrene tissue culture flasks at 37°C in a 5% CO_2_ incubator.

### 2.4 Adherence Test

Bacterial adherence to epithelial cells was assessed as described previously [25]. The cells were seeded in a 24‐well plate (Corning, NY, USA). To induce polarization, Caco‐2 cells were cultured for at least 2 weeks, with the medium (2% FBS) changed twice weekly. Other cell lines were used upon reaching confluence. Before the experiment, the medium was replaced with culture medium containing d‐mannose (1%, w/v) to prevent bacterial type I pili adhesion [26]. Moreover, bacteria were cultured overnight, and the optical density of the medium at 600 nm (OD600) was measured to estimate colony‐forming units (CFUs). Bacteria suspensions in culture medium were used to infect cells at a bacteria‐to‐cell ratio of 10:1. Co‐cultures were incubated for 3 h. The monolayers were washed with PBS, fixed with methanol, stained with Giemsa, and photographed under a BX53 light microscope (Olympus, Tokyo, Japan) to monitor the adherence of the bacteria to the cells. To quantify the bacteria attached to the cells, CFUs from each well were calculated. After bacterial inoculation and microscopic evaluation, the cells were lysed with 1% Tween‐80 and plated on LB plates after dilution to count the colonies. Antibody inhibition experiments were performed by adding each antiserum (1:5000 dilution) 3 h before bacterial inoculation.

The results were statistically analyzed using Prism software (GraphPad ver. 8.4.3). Differences in CFU were assessed using a one‐way ANOVA and Tukey's multiple comparison tests.

### 2.5 Antisera Against Major Adhesin Subunits (FayG1 and FayG2) of F4_O169_

Rabbit polyclonal antisera against FayG1 and FayG2 were generated from respective recombinant proteins as described previously [27].

### 2.6 Western Blot Analysis

Each bacterial strain was grown overnight at 37°C in 1 mL of TSB liquid medium. Cultures were centrifuged at 10,000 rpm for 5 min, and the pellets were collected and boiled in 200 μL of sample buffer containing 0.1 M Tris‐HCl (pH6.8), 4% (*w*/*v*) sodium dodecyl sulfate (SDS), 12% (*v*/*v*) 2‐mercaptoethanol, and 20% (*v*/*v*) glycerol. A total of 0.25 μL of each sample was loaded for electrophoresis, while recombinant FayG1 and FayG2 proteins were loaded at 400 ng per well. SDS‐polyacrylamide gel electrophoresis was performed using 12% SDS‐polyacrylamide gels. Protein bands were transferred from the gels to the polyvinylidene difluoride membrane using the iBlot2 Dry Blotting System (Thermo Fisher Scientific, MA, USA). Membranes were blocked with Tris‐buffered saline with 0.02% Tween‐20 (Fujifilm Wako Pure Chemical Corp.) (TBS‐T) containing 5% skim milk for 1 h, followed by incubation with primary antisera (αFayG1 or αFayG2) at a dilution of 1:200 each for 1 h. After three washes using TBS‐T, membranes were incubated with a secondary antibody (anti‐rabbit IgG, HRP‐linked whole Ab donkey; Cytiva, Tokyo, Japan) at a dilution of 1:12,500 for 1 h. After three washes, identical to those performed with the primary antisera, peroxidase bound proteins on the membrane were detected with ECL Prime Western blot analysis System (Merck KGaA, Darmstadt, Germany) and visualized using a ChemiDoc molecular imager (Bio‐Rad Laboratories, CA, USA).

### 2.7 In Silico Surveillance of *fayG1* and *fayG2* Genes

In silico surveillance of two adhesin subunits of F4_O169_ (FayG1 and FayG2) was conducted by using genome sequences from the Bacterial and Viral Bioinformatics Resource Center database [28]; in detail, a total of 11,396 strains were retrieved, comprising 3006 bovine, 2088 porcine, and 6302 human isolates (registered up to October 31, 2023). Amino acid (AA) sequences of open reading frames encoding > 50 AAs were created using getorf in the EMBOSS package [29]. Subsequently, the AA sequences were subjected to a BLASTp search using FayG1 and FayG2 sequences as queries, with 1E−20 as the E‐value threshold. The truncated sequences were filtered by manual curation.

For phylogenetic tree reconstruction, the identified sequences and controls were trimmed using SignalP v. 5.0 [30] to remove signal peptides and then aligned using MAFFT v7.490 [31], followed by polishing with trimAL v1.4. rev15 [32] was used to remove positions with gaps greater than 80%. The resulting alignments were used to construct the NJ trees using MEGAX [33] with the JTT model [34] and 500 bootstrap iterations. The newly obtained tree file was visualized using iTOL version 6.9.1 [35].

Multilocus sequence types (MLST) and O‐antigen types of six strains possessing *fayG1/G2* genes were determined by using MLST v2.0.9 [36] and SerotypeFinder v2.0.1 [37], both provided by the Center for Genomic Epidemiology web service.

## 3 Results

### 3.1 Adhesion to Human Epithelial Cells

**Figure 1:**
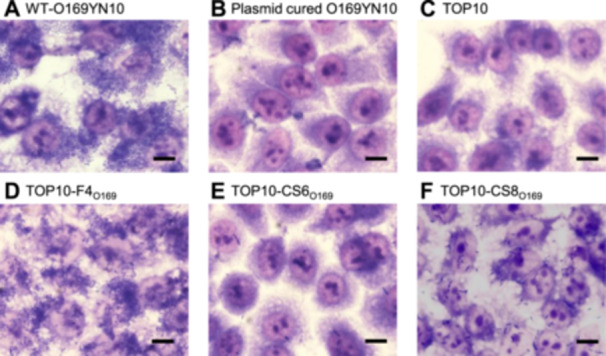
Adhesion of *Escherichia coli* laboratory strains expressing recombinant CFs of pEntYN10 to human epithelial cells. Adhesion images of wild‐type O169YN10 (A), plasmid‐cured O169YN10 (B), parental TOP10 (C), TOP10/F4_O169_ (D), TOP10/CS6_O169_ (E), and TOP10/CS8_O169_ (F) to HEp‐2 cells. Scale bars, 10 μm.

**Figure 2:**
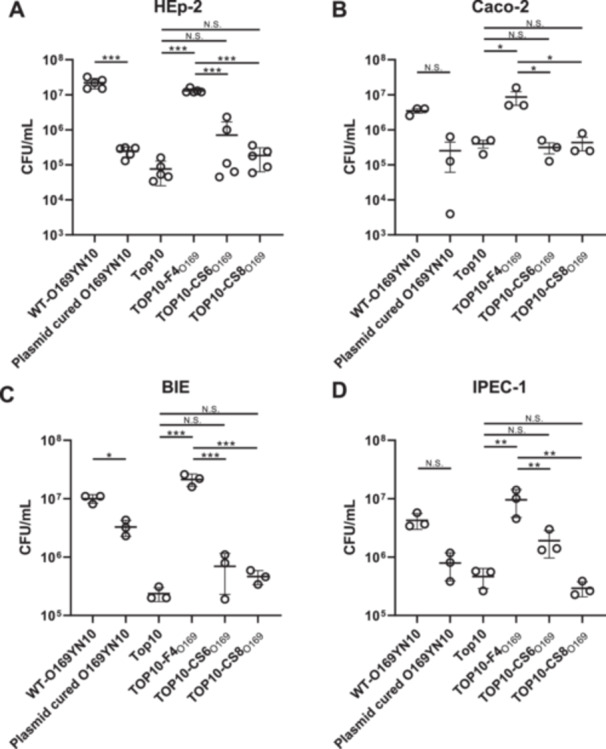
Adhesion of each CF of pEntYN10 to porcine and bovine intestinal epithelial cells. CFUs of bacterial strains and derivatives adhered to HEp‐2 cells (A, *n* = 5), Caco‐2 cells (B, *n* = 3), BIE cells (C, *n* = 3), and IPEC‐1 cells (D, *n* = 3). Values are represented by individual plots and mean ± standard deviation (SD). Statistical analysis was performed with one‐way ANOVA and Tukey's multiple comparison tests. ***p < 0.001.

To clarify which of the three CFs mediated by pEntYN10 played a pivotal role in unique aggregative adhesion to human cells, a laboratory strain of *E. coli* (TOP10) was transformed with plasmid pSTV28‐based recombinants carrying the respective CFs. Wild‐type O169YN10 cells exhibited cohesive adhesion to HEp‐2 cells (Figure 1A) and failed to adhere when the plasmid was cured (Figure 1B). The laboratory strain TOP10, which showed no adherence (Figure 1C), was transformed with the recombinant plasmid F4_O169_ (TOP10/F4_O169_) and adhered to human HEp‐2 cells, similar to the O169 strain (Figure 1D). Strains transformed with recombinant plasmids carrying the variant of CS6_O169_ and CS8_O169_ (TOP10/CS6_O169_ and TOP10/CS8_O169_) adhered very slightly to HEp‐2 cells (Figures 1E,F). The number of bacteria recovered from HEp‐2 cells also confirmed these observations (Figure 2A). The O169YN10 WT showed higher CFUs in HEp‐2 cells, whereas the O169 non‐plasmid showed significantly lower CFUs. Among the TOP10 strains carrying each operon, TOP10/F4_O169_ showed significantly higher CFUs than did the parental strain, whereas TOP10/CS6_O169_ and TOP10/CS8_O169_ showed nonsignificant but higher CFU trends. Adhesion tests using Caco‐2 cells, which are human intestinal epithelial cells, confirmed the adhesive ability of F4_O169_, consistent with the results obtained using HEp‐2 cells (Figures 2B and S1A).

### 3.2 Adhesion to Porcine and Bovine Epithelial Cells

Because F4 is well known as a prominent virulence factor of *E. coli* causing porcine diarrhea [38, 39], and F4_O169_ adhesins were serologically positive in bovine and porcine [27], the adhesive properties of the F4_O169_ against porcine and bovine intestinal epithelial cells were suggested. TOP10/F4_O169_, TOP10/CS6_O169_, and TOP10/CS8_O169_ cells were inoculated into IPEC‐1 and BIE cells as in the HEp‐2 experiments. The parental strains O169YN10 and TOP10/F4_O169_ adhered well to BIE (Figures 2C and S1B) and IPEC‐1 cells (Figures 2D and S1C), whereas neither TOP10/CS6_O169_ nor TOP10/CS8_O169_ adhered abundantly to these epithelial cells.

### 3.3 Inhibitory Effect of Anti‐FayG1 and FayG2 Sera on the Adhesion

**Figure 3:**
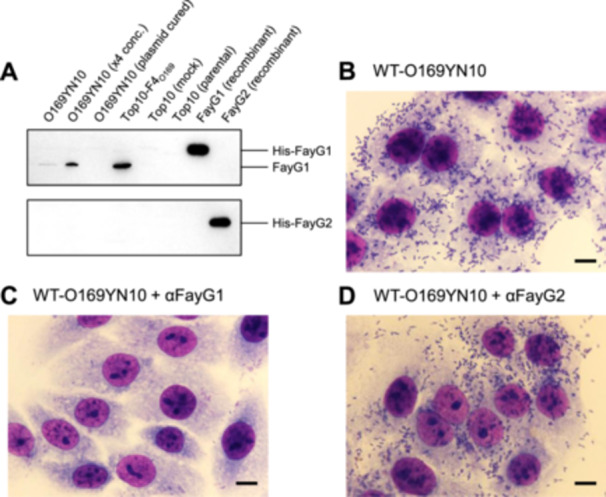
Detection of FayG1 and FayG2 and their role in bacterial adhesion to epithelial cells using specific antisera. Western blot analysis is performed using αFayG1 (top panel) and αFayG2 (bottom panel) antisera (A). HEp‐2 cells are infected with strain O169YN10 under different conditions: without treatment (B), in the presence of αFayG1 antiserum (C), and in the presence of αFayG2 antiserum (D). Scale bars represent 10 μm.

To confirm the roles of the two adhesins, FayG1 and FayG2, which comprise the unique operon structure of F4_O169_, antibodies against each recombinant protein were prepared. Western blot analysis confirmed the presence of FayG1 in the original strain, while FayG2 was not detected (Figure 3A). When added to HEp‐2 cells before infection with O169YN10, bacterial adherence diminished with antiserum against FayG1 (Figure 3B,C). Despite inhibition by antiserum against FayG1, antiserum against FayG2 did not affect the adhesive phenotype of the bacilli (Figure 3D).

### 3.4 In Silico Surveillance of the Adhesins

The in vitro adherence assays demonstrated the ability of F4_O169_ to mediate cell adherence across multiple host species (Figure 2). This observation prompted us to investigate the distribution of F4_O169_ adhesin genes among the strains isolated from multiple host species.

**Figure 4:**
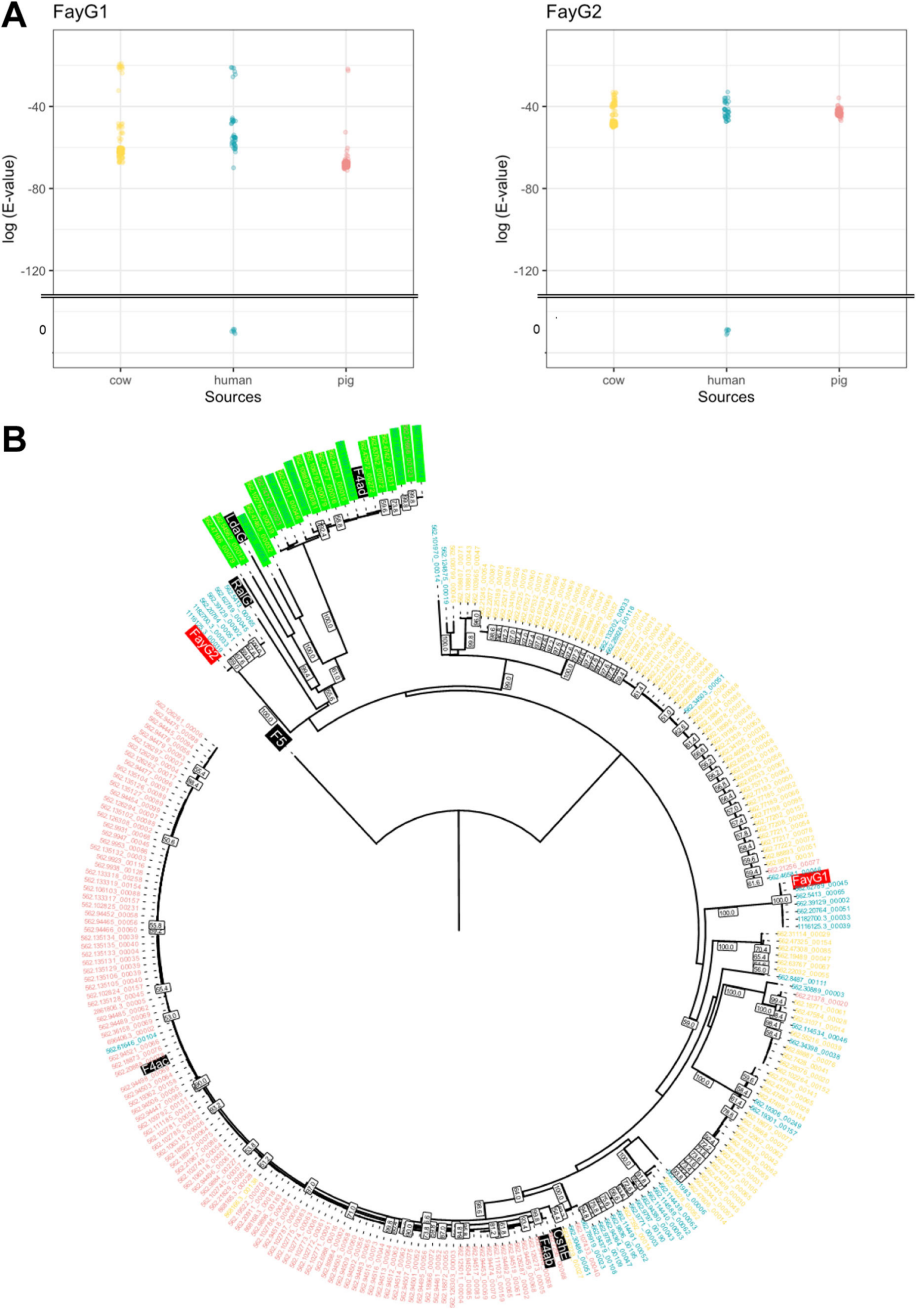
(A) Distribution of E‐values for FayG1 and FayG2 homologs identified by BLASTp search to 11,396 *E. coli* genome sequences isolated from humans, bovine, and porcine. Six sequences from human strains identical to both FayG1 and FayG2 of F4_O169_ were plotted at the zero of E‐values. (B) A phylogenetic tree of AA sequences identified by BLASTp search using FayG1 and FayG2 queries. The labels represent genome IDs and contig numbers as assigned in the BV‐BRC database. The colors indicate the hosts, corresponding with (A). Twenty sequences were only found in the FayG1 homologs search, whose labels are highlighted in light green. Seven control sequences (FaeG from F4ab, F4ac, and F4ad; CshE from CS13; RalG from Afr2; LdaG from Lda; and FanC from F5) and two FayG1/FayG2 are included in this tree. FanC was determined as a root. The bootstrap values were described at the side of each branch when they were over 50.

Our in silico surveillance revealed a low prevalence of adhesins with a high homology to FayG1 and FayG2 across the examined genome sequences (Figure 4). When sequences with E‐values less than 1E−20 were extracted, FayG1 homologs were found in 45 sequences from 6302 human strains, 107 from 3006 bovine strains, and 115 from 2088 porcine strains (Figure 4A). For FayG2, all hit sequences overlapped with those of FayG1: 39 from humans, 95 from cattle, and 113 from pigs. Almost all homologs detected in this search had E‐values greater than 1E−70 (corresponding to identity in AA sequences of less than 45%) for FayG1 and more than 1E−50 (corresponding to less than 39%) for FayG2 for the strains from all three hosts. Among the 11,396 *E. coli* genomes analyzed, six human‐derived strains had genes identical to those of *fayG1* and *fayG2* but exhibited variable MLST profiles and O‐antigen types (Table 2). The AA sequences of FayG1 and FayG2 were also similar, with mutual E‐values lower than 1E−50.

**Table 2:** *E. coli* strains possessing FayG1 and FayG2 found in a genome database (BV‐BRC).

Name of strain	MLST	Collection year	Isolation Country	Genome ID^a^	Reference
214‐4	398	1974	Mexico	562.39129	Hazen et al. [40]
MOD1‐EC1556	316	1997	Guinea‐Bissau	562.20764	Gangiredla et al. [41]
2845650	182	2008	Bangladesh	1116125.3	unpublished
KTE101	357	2010	Denmark	1182700.3	unpublished
CE516	1490	2010	China	562.5413	unpublished
BS28R‐B	182	2019	Switzerland	562.62789	unpublished

^a^ ID number assigned in the genome database BV‐BRC [27].

## 4 Discussion

O169YN10 has been shown to possess three adhesin homologs (CS6_O169_, CS8_O169_, and F4_O169_) with the enterotoxin STp [24]; however, the clear role of these adhesin homologs in adherence to host cells remain unclear. This study showed that F4_O169_ fimbriae exhibit a broad affinity for human, bovine, and porcine epithelial cells, indicating that the operon provides host‐versatile adhesive potential to bacteria. For attachment to human cells, CS6 is well known as a typical CF; however, the variant CS6_O169_ contributed less to attachment to cells in this study. These results indicate that the F4_O169_ fimbria can function as a single, inter‐host shuttle adhesin responsible for the infection of multiple animal species rather than as an adhesin conferring specific host targeting. However, it should be noted that this study did not investigate the potential effects of epithelial cell polarity or surface molecule distribution on the adhesion ability of F4_O169_. Moreover, microbiota composition and mucosal secretions were not assessed, which should be elucidated to understand their potential impact under in vivo conditions. Despite these limitations, multi‐host adherence shown by F4_O169_ may be significant evidence of its efficacy as a zoonotic or anthropogenic factor that can cause inter‐host transmission. These findings highlight the potential of broad‐host‐range adhesion systems to contribute to cross‐species infections and suggest that such mechanisms should be considered in strategies for controlling ETEC transmission.

It is important to understand how the major adhesin subunits encoded in the F4_O169_ operon are related to the adhesive function in various host epithelial cells to uncover their molecular basis as a virulence factor. Adhesion inhibition assays using antibodies confirmed that FayG1 plays a more significant functional role (Figure 3). The inhibition of bacterial cell adherence by the αFayG1 antibody suggests that an immune response against the fimbrial subunit could confer protective immunity, highlighting its potential as an effective vaccine target. Although the FayG2 protein was not detected under in vitro conditions, this absence may reflect regulatory factors affecting its translation or stability, warranting further investigation. The exact role of FayG2 remains unclear, but it may act as a minor adhesin subunit. Indeed, CU fimbriae of the *K* class frequently possess multiple minor adhesin subunits [8]. Further studies focusing on the individual functions of FayG1 and FayG2 will contribute to a deeper understanding of fimbrial‐mediated adhesion mechanisms.

In silico analysis demonstrated that a few adhesin genes highly homologous to *fayG1* and *fayG2* were identified in *E. coli* strains from various origins, including humans, bovine, and swine. This indicates that this atypical CU fimbria is unlikely to have been widely disseminated and established across multiple host species despite in vitro adherence. Interestingly, sequences identical to *fayG1/G2* were found in only six human‐derived strains. Although these strains originated from geographically and temporally disparate sources (Table 2), this is consistent with the fact that O169 ETEC strains have been reported in various regions. However, serological studies have shown a sufficiently high percentage of antibody‐positive individuals in these animals [27], suggesting that strains carrying these adhesins may be isolated through more extensive surveillance, including a broad range of bacterial species, in the future. Furthermore, while typical F4 fimbriae are known to exhibit host specificity, adhering predominantly to porcine cells [42], adhesive targets of other homologous fimbriae have not been evaluated in detail. Importantly, the broad‐spectrum adhesive properties of F4_O169_ may indicate its unique feature. Future investigations into whether other homologous fimbriae share this broad‐spectrum adherence could provide insights into the evolutionary and functional diversity of CU fimbriae.

Remarkably, the reported adhesins among the *K* class of CU fimbriae, F4 (K88), CS13, CS23, and Lda, exhibited low sequence similarity to FayG1 and FayG2, indicating their distinct evolutionary relationships (Figures 4 and Figure S1). One possible reason is that F4_O169_ may have been acquired from different bacterial species via horizontal gene transfer. Supporting this hypothesis, previous studies have reported sequences homologous to these adhesins in different genera of *Enterobacteriaceae*, such as *Salmonella enterica* and *Erwinia* [24]. As of December 2024, a BLAST search of the NCBI database revealed that the homologous sequences of FayG1 and FayG2 remain unchanged compared to those reported in our previous study [24] (data not shown). Further understanding of the distribution and diversity of CU fimbriae across multiple bacterial species may shed light on the host adaptation strategies of Enterobacteriaceae.

In summary, we showed a broad host range of the adherence function of a new fimbria similar to that of F4 (K88), while lower functions of the other adhesins were encoded on pEntYN10, a virulence plasmid of ETEC O169YN10. It seems that strains possessing fimbriae might be transmitted from domestic animals to humans (zoonotic) or from humans to domestic animals (anthropogenic) using a unique fimbria, F4_O169_, and its homologs, owing to multi‐host‐adherence. In silico surveillance of *E. coli* strains revealed that the fimbriae similar to it were almost not found, suggesting its origin might be different bacterial species based on horizontal transmission. Notably, this multi‐host adaptive adhesin has been isolated without specific regionality, although at a low frequency.

## Author Contributions

Y.N. and T.W. designed the study. H.I., Y.T., D.Z., M.I., and Y.O. conducted the experiments. H.A. and W.Z. contributed to the preparation of the cultured cells for the experiments. H.I. and Y.T. analyzed in vitro experimental data. H.I. conducted in silico surveillance of the adhesin subunits. Y.Y., T.T., and E.K.N. contributed to the preparation of critical reagents/genetic tools used in the study, especially in the preparation of antigens. H.I., Y.T., Y.N., and T.W. drafted the manuscript. All authors have read and approved the contents of the manuscript.

## Disclosure

The authors have nothing to report.

## Conflicts of Interest

The authors declare no conflicts of interest.

## Supporting information

Supporting information.

Supporting information.

## Data Availability

The data that support the findings of this study are available from the corresponding author upon reasonable request.
