# Supplementary material for: An Atypical Kappa‐Class Chaperone‐Usher Fimbriae of a Human Enterotoxigenic Escherichia coli Strain Shows Multi‐Host Adherence and Distinct Phylogenetic Feature
Source: Microbiol Immunol. 2025 Mar 2;69(5):270–9. doi: 10.1111/1348-0421.13208 (PMC12050912; doi:10.1111/1348-0421.13208)
Supplement: Supplementary file 1 — Supporting information. [file MIM-69-270-s002.pdf]

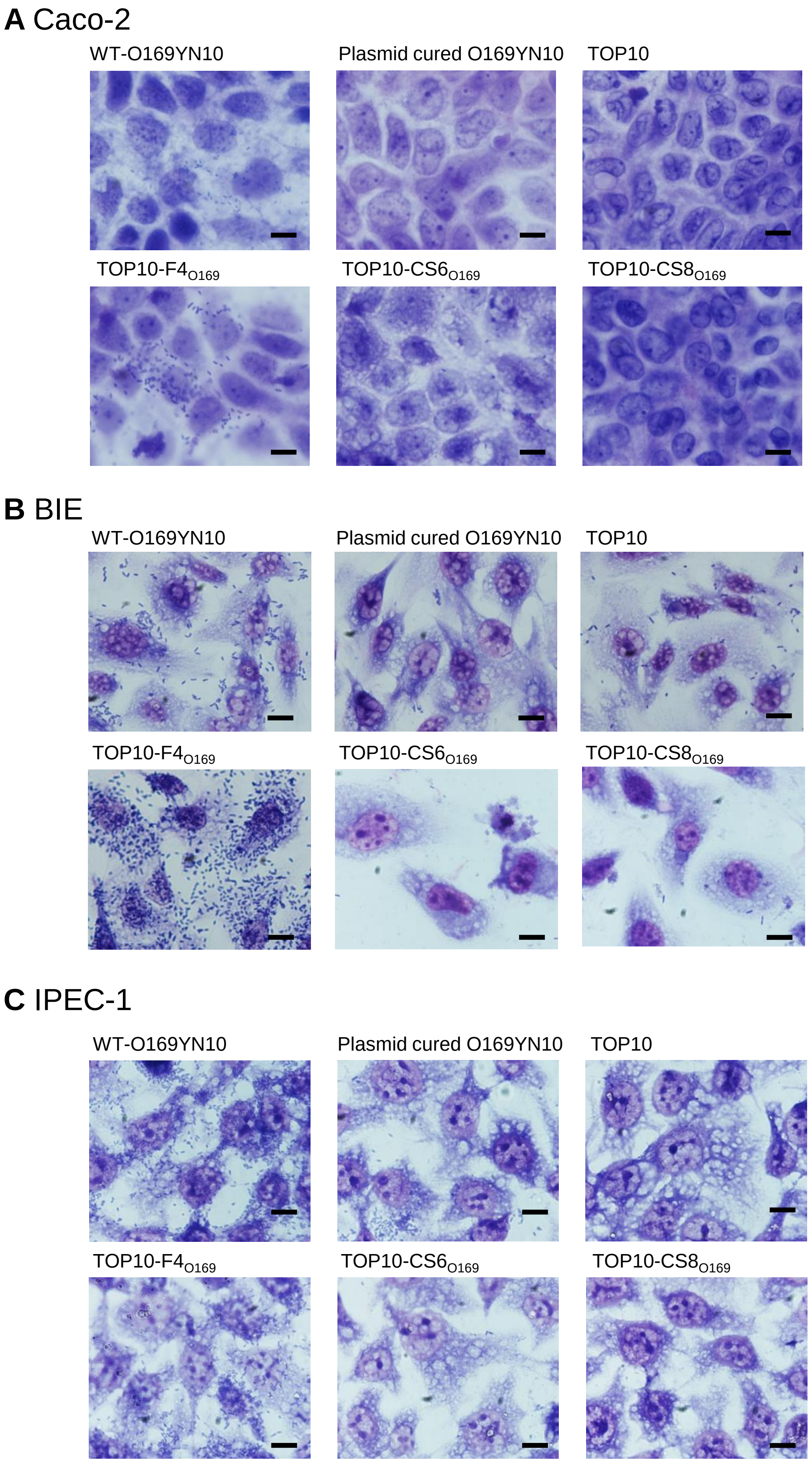

Supplementary Materials

Fig. S1 Adhesion of *Escherichia coli* laboratory strains expressing recombinant CFs of pEntYN10 to epithelial cells of humans (Caco-2), bovine (BIE), and porcine (IPEC-2). Adhesion images of wild-type O169YN10, plasmid-cured O169YN10, parental TOP10, TOP10/F4$_{O169}$, TOP10/CS6$_{O169}$, and TOP10/CS8$_{O169}$ to cells. Scale bars, 10 μm.
